# Do Catholics Listen to Sermons?

**DOI:** 10.1007/s10943-020-01055-y

**Published:** 2020-07-09

**Authors:** Anna Maria Noworol OV, Witold Ostafiński

**Affiliations:** Uniwersytet Papieski Jana Pawła II w Krakowie (John Paul II Pontifical University in Kraków), Kraków, Poland

**Keywords:** Sermon, Listenability of sermons, Catholics

## Abstract

The article discusses the issue of listening to sermons in Catholic churches, in Poland. In the first part, it presents the author’s CKSK method, measuring whether and how Catholics listen to sermons from a theological and psychological point of view. The study was conducted on 130 people aged 16–92 years, in three Roman Catholic churches, in natural conditions, non-experimental. The research on the audience of sermons shows that Catholics usually listen to sermons in a fragmentary, medium-attentive way. The results indicate that the listenability of sermons is gender independent. It does not depend also on whether the parish is urban or rural, so it depends on other features and factors. However, listening to sermons varies from one age group to another. The study confirmed also the lack of correspondence between the declared listenability of sermons and their actual listening; only 7.84% of the variance of the declared listenability of sermons is explained by the variance of the actual listening to sermons.

## Introduction

According to the tradition of the Roman Catholic Church, sermons are preached at Sunday and holiday masses and in other circumstances. Priests preach according to the command of the Lord Jesus: “ Go into all the world and preach the Good News to everyone” (Mark 16:15). The purpose of preaching is to communicate religious and moral contents, for preaching is the religious language which proclaims the truth of God to the faithful, or to those converted to faith, with all the duties it imposes on men and women (Pilch 1958). However, besides the preparation of the preacher, especially the preparation of the content, and the preaching of the sermon, that the content might be implemented by the people, there is a need for someone to listen to the sermon.

The question of whether Catholics listen to sermons is interesting not only for priests. The answer to this question is psychologically conditioned, because in interpersonal communication listening plays a fundamental role. The method CKSK of examining the audience whether Catholics listen to sermons, created by the authors of this article, is a method measuring one of the aspects of interpersonal communication, which is the audience. After the method was developed, its authors conducted research, the results of which suggest (95% confidence interval) what is the listenability of sermons in Poland.

## Method

### Description of the CKSK

The scientific goal of the CKSK project “Do Catholics Listen to Sermons” was to construct a method to measure whether the faithful listen to sermons. Because of the lack of homiletic and psychometric measurement tools, a new one was created (CKSK). The CKSK is based on short-term memory, which stores information for up to 30 min (Zimbardo and Ruch 1988; Klein and Kolb 2008; Atkinson 1995). This method consists in arranging 5 test questions for a given sermon, with 3 choices. Only one answer is consistent with the content of the sermon, the others are not, however, created in such a way that they can be chosen by people who ticked “at random” (i.e. people who did not listen to the sermon, so they do not know the correct answer, consistent with the content of the sermon; however, having a question before them, they try to mark an answer; every wrong answer is somehow probable, so that without knowing the content of the sermon each answer can be chosen with the same probability). In the command, listeners are asked to select one answer, the one that matches the content of the sermon heard just now. The faithful receive question cards immediately after the blessing at the end of the Mass and are asked to fill them in immediately (i.e. immediately upon receipt, but with no time limit (it usually takes a few minutes to fill in). The theoretical foundations of the research method are the same as those of the interviewers working after symposia or lectures (e.g. Cannel and Kahn 1965). Thanks to such a structure, it is a universal method that can be used for researching adults of all ages, states, education; it does not require knowledge of theology, the ability to tell stories, or other skills that are needed when students are asked to report on a sermon, or even to tell a fragment of it.

In addition, the method tries to eliminate (as far as possible) the influence on the results of the research of the factors that influence the processes of remembering information; both open questions and long-term memory studies (after more than 30 min) are strongly influenced by these factors. When one examines the memory of a sermon in such a way that the person must remember, i.e. extract all the necessary information contained in the memory track (i.e. answers open questions) by himself (Maruszewski 2000), one has to do with memorizing arguments; this in turn may be connected with long-term memory. Attitudes also have an impact on memory, i.e. you remember the content in accordance with your own attitudes better, and worse, what is incompatible with them. And importantly, it is faster to forget what is incompatible with attitudes (Mika 1987), so to conduct research focused on listenability, and not on memory as such or researching attitudes, the most effective is the choice test (closed-ended, single-choice questions) performed properly immediately after listening.

In addition, the sermon should influence listeners at cognitive (they learn new information, religious and moral knowledge), behavioural (the content they hear is transferred into their own practical actions), and emotional (Schwarz 1993) levels. Emotions influence, among others, the attention of listeners (e.g. someone bored or nervous about mistakes stops listening), and the motivation to listen (e.g. people are not willing to listen to someone they do not like, and if they listen, their judgment will be more critical); because affect (emotions and moods) influence perception and memory availability, and emotions are both the result of data processing (people stop listening or start listening) and the effect of information processing (Clore 1998). The emotional reactions of the listeners may affect the audience’s listenability of the sermon (the end of listening or the beginning of listening), but similarly other factors may cause listening to take place from a certain moment (“getting involved”) or until a certain moment (“getting out”), such as fatigue, duration, incomprehensibility, and reverie. Thus, the CKSK study covers the audience’s listenability of the initial, middle, and final part of the sermon (results for individual questions) in an objective manner, since it assesses listenability regardless of its cause.

The CKSK method is constructed in such a way as to really examine the audience’s listenability of the sermon, and not the memory of the listeners, their emotions, attitudes, or other factors. The CKSK examines the listenability of the sermon in contrast to other authors’ research, which assessed the subjective perception of the sermon, not its listenability. First of all, CKSK does not measure cognitive reactions of listeners, i.e. learning new content, because researching this aspect would require research related to long-term memory. In addition, the CKSK method is designed for research in natural conditions, i.e. people who attended a Mass and listened to the sermon at the Mass, and at the Mass in a typical church, listeners have different ages, levels of education and religious knowledge, degree of piety, and many other characteristics and attitudes that influence whether something is new for them or not.

Moreover, attitudes are relatively constant (Aronson et al. 1997), so a possible change in attitude is usually a complex and long-lasting process, and although a single message could influence a change in attitude, in order to objectively assess such a change, it would be necessary to assess a person’s attitude to a given issue both before and after the message (i.e. before and after listening to the sermon).

Secondly, remembering arguments, understanding the sermon, and assimilation of content are essentially long-term memory, if they concern the transfer of sermon content to the affairs of one’s own life; research into these factors would require a significant time lag and a method comparing a given aspect of a person’s life before and after listening to the sermon. It seems that such research could be reliably carried out only with the use of the case study method.

Thirdly, the evaluation of a sermon is a very subjective matter, which may not coincide with the quality of the sermon (the evaluation of this aspect objectively depends, among other things, on knowledge of theology), nor with its message (the evaluation depends, among other things, on the state of life, piety), nor even with the listenability (e.g. listeners say “I liked it”, but they do not know what the sermon was about. Please note that if this happens after a longer period of time, e.g. a few days after listening to the sermon, it is only a matter of memory, but if the listeners speak in this way immediately after Mass, it is usually possible to conclude that they are not listening attentively.) Thus, the CKSK method is abstract from these aspects, and although it has a simple structure, it retains as much objectivity as possible.

The research was carried out in relation to three sermons. The three sermons that the respondents listened to were very different (e.g. there was an uneven substantive level of the sermons). One was very well prepared, going from the Bible to life. The second sermon was very short (the priest spoke about 3–4 min), and contained too little religious content; it was actually a secular speech, which cannot be entirely called a sermon (because if there is no religious content, the speech loses its name as a sermon; Pilch 1958). However, people could not expect to hear what they would hear, because many preachers sometimes preach better and sometimes worse, so there was no need to skip this sermon in their research. The third was a typical case of various repetitions of the Gospel (e.g. repetition of the same fragments with one’s own words; a method criticized by homilies); it was an average sermon, although it contained religious content and moral message, but it was poorly elaborated. Probably this variety of sermons, to which the CKSK method was applied, made it possible to find a more reliable average audience for sermons in Poland, because even if there are dioceses with a larger and smaller percentage of faithful practitioners, in every diocese there are good, inadequate, and average sermons preached; thus the research refers to all kinds of sermons (i.e. the possibilities of sermons presented, from the side of the preacher).

The CKSK results were prepared on the basis of the division of the listeners into six groups (according to the number of correct answers, i.e. according to the content of the sermon heard a moment ago): *Accurate Listeners* (i.e. those who answered all questions correctly, scored 5 points), *Fairly Accurate Listeners* (scored 4 points), *Average Listeners* (3 points), *Low Accuracy Listeners* (2 points), *Almost Non*-*Existent Listeners* (1 point), *Non*-*Listeners* (i.e. gave only wrong answers, and scored 0 points).

### Participants and Course of Research

The research was conducted on 130 people (82 women and 48 men), aged 16–92 years, *M *= 47.5, SD = 22.9 in three churches (Roman Catholic Holy Masses, sermons preached by priests), namely 2 city parishes and 1 rural parish, in natural conditions (i.e. the study was conducted on the participants of Mass who were asked to answer the questions of the CKSK only after hearing the sermon, just after the blessing for sending) in 2018.

## Results

### Number of Good and Bad Answers to Each Question

Correct answers (consistent with the content of the sermon) indicate listening to the sermon. The sequence of the questions relates to its phases, from its beginning to its end (Table 1).

**Table 1:** Number of listeners (score = 1) and non-listeners (score = 0) at the particular phases of the sermon (question 1 = introductory part; question 3 = the middle; question 5 = the final part); *N *= 130

Question	Scores	Number	Percentile
1	0	55	42.31
	1	75	57.69
2	0	47	36.15
	1	83	63.85
3	0	45	34.62
	1	85	65.38
4	0	36	27.69
	1	94	72.31
5	0	55	42.31
	1	75	57.69

As can be seen in Table 1, almost 58% of people listened to the beginning of the sermon (question 1, score = 1) and 42% of people did not (question 1, score = 0). Next, almost 64% of people listened to the following part of the sermon (question 2, score = 1) and 36% did not (question 2, score = 0). The middle part of the sermon was listened to by about 65% of the participants (question 3, score = 1) and 35% did not (question 3, score = 0). Afterwards, nearly 72% of persons listened to the subsequent part of the sermon (question 4, score = 1) and 28% did not (question 4, score = 0). Finally, almost 58% of the participants of the Holy Mass listened to the end part of the sermon (question 5, score = 1) and 42% did not (question 5, score = 0).

**Fig. 1:**
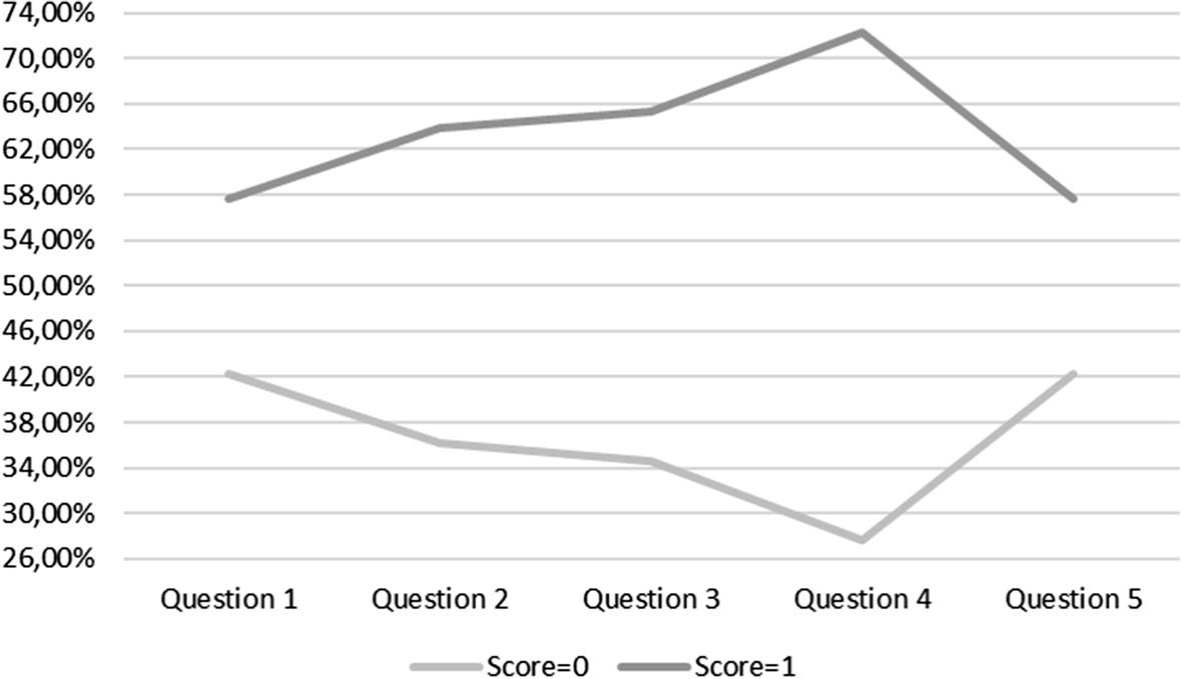
Correct answers (consistent with the content of the sermon, score = 1) indicate percentiles of participants’ number listening to the sermon. The sequence of the questions (1–5) relates to its phases, from its beginning to its end. Score = 0 indicates percentiles of those who did not listened to the sermon

The data in Table 1 shows that the most number of participants listened to the middle part of the sermons (questions 2, 3, 4) with the maximum (72.31%) at the phase 4 (Fig. 1).

It can be deduced from this that some people start listening not from the very beginning, and some “switch off” after a while. It is also worth noting that of the sermons used in the study, one was well prepared, passing from the Bible to everyday life, quite long, interesting, and neither original nor exaggeratedly linguistically sophisticated. After this sermon, almost all (93%) answered the last question correctly.

These results confirm the assumption of the research method created, its foundation on the action of short-term memory (apart from the research time, if one were to deal with long-term memory, other parts of the sermon would most probably be remembered (e.g. Aronson et al. 1997). The results also confirm the experience known from the listeners that some people start to listen to the sermon only after some time, so it is good that the sermon is not too short. On the other hand, there are people who stop listening after some time, often bored with the uninteresting content of the sermon, so the sermon should also be understandable, with the simultaneous attention that it should not fall into banality, because the language used in the sermon influences the understanding between the preacher and the listeners (Matuszczyk 2003).

### Sum of Correct Answers (Listenability by Participants)

The question arises as to how many people in total listened exactly to the sermon, that is, how many listeners could be classified to each of the six groups defined from *Accurate Listeners to Non*-*Listeners*.

As can be seen in Table 2, which presents the results for persons (i.e. the results showing how the persons listen to the sermon in total, and not what is the listenability of particular phases of the sermon, i.e. number of correct answers for particular questions), only about 9% of *Non*-*Listeners* (not a single correct answer) and exactly 20% of *Accurate Listeners* (who answered all questions correctly). It can be seen that most people listened to the sermon partially (not from the beginning, not to the end, or fragmentarily). There were over 28% of *Fairly Accurate Listeners*, 22% of *Average Listeners*, 16% of *Low Accuracy Listeners* (who listened not very attentively) and almost 4% of *Non*-*Existent Listeners* (who listened very carelessly and selectively).

**Table 2:** Listenability by participants, *N *= 130

Sum of correct answers	Number	Cumulative number	Per cent	Cumulative per cent
0-*Non*-*listeners*	12	12	9.23	9.23
1-*Almost non*-*existent listeners*	5	17	3.85	13.08
2-*Low accuracy listeners*	21	38	16.15	29.23
3-*Average listeners*	29	67	22.31	51.54
4-*Fairly accurate listeners*	37	104	28.46	80.00
5-*Accurate listeners*	26	130	20.00	100.00

### Sum of Correct Answers in Age Ranges

The results of listening to sermons in the age range 16–39 for the sum of the participants’ points are presented in Table 3.

**Table 3:** Listening to sermons from 16 to 39 years of age, *N *= 51

Sum of correct answers	Number	Cumulative number	Per cent	Cumulative per cent
0-*Non*-*listeners*	3	3	5.88	5.88
1-*Almost non*-*existent listeners*	0	3	0.00	5.88
2-*Low accuracy listeners*	5	8	9.80	15.69
3-*Average listeners*	14	22	27.45	43.14
4-*Fairly accurate listeners*	16	38	31.37	74.51
5-*Accurate listeners*	13	51	25.49	100.00

As can be seen in Table 3, among people in this age group, most people listen to the sermon quite accurately (over 31% of *Fairly Accurate Listeners*) and on average attentively (over 27%). There were also no people in the group of *Almost Non*-*Existent Listeners,* although nearly 6% did not listen at all.

As can be seen in Table 4, among middle-aged people, more than 88% listen to sermons accurately, in groups from *Average Listeners* to *Accurate Listeners*, which is only 4% more than among young people (Table 3). Those who have not listened at all account for almost 6%.

**Table 4:** Listening to sermons from 40 to 59 years of age. *N *= 34

Sum of correct answers	Number	Cumulative number	Per cent	Cumulative per cent
0-*Non*-*listeners*	2	2	5.88	5.88
1-*Almost non*-*existent listeners*	1	3	2.94	8.82
2-*Low accuracy listeners*	1	4	2.94	11.76
3-*Average listeners*	8	12	23.53	35.29
4-*Fairly accurate listeners*	13	25	38.24	73.53
5-*Accurate listeners*	9	34	26.47	100.00

As can be seen in Table 5, among the elderly, most people listen to the sermon not exactly (nearly 27% of *Low Accuracy Listeners*) and what is more, over 12% of people did not listen at all, and only 10% of people were *Accurate Listeners*.

**Table 5:** Listening to sermons from 60 to 79 years of age. *N *= 30

Sum of correct answers	Number	Cumulative number	Per cent	Cumulative per cent
0-*Non*-*listeners*	5	5	12.50	12.50
1-*Almost non*-*existent listeners*	2	7	6.67	23.33
2-*Low accuracy listeners*	8	15	26.67	50.00
3-*Average listeners*	6	21	20.00	70.00
4-*Fairly accurate listeners*	6	27	20.00	90.00
5-*Accurate listeners*	3	30	10.00	100.00

As can be seen in Table 6, among people over 80 years of age, most people listen to sermons not exactly (over 46% of *Low Accuracy Listeners*). However, it should be taken into account that in this age group also short-term memory may be slightly weaker, and this group was not so well represented by the survey participants. Nevertheless, interesting patterns have manifested themselves anyway, namely about 27% of people were *Average, Fairly* and *Accurate Listeners,* and the same per cent of participants were *Almost Non*-*Existent Listeners and No*-*Listeners.* These results confirm the correct structure of the research method, because based on short-term memory, elderly people were also able to give the correct answers quite easily.

**Table 6:** Listening to sermons from 80 to 92 years of age, *N *= 15

Sum of correct answers	Number	Cumulative number	Per cent	Cumulative per cent
0-*Non*-*listeners*	2	2	13.33	13.33
1-*Almost non*-*existent listeners*	2	4	13.33	26.67
2-*Low accuracy listeners*	7	11	46.67	73.33
3-*Average listeners*	1	12	6.67	80.00
4-*Fairly accurate listeners*	2	14	13.33	93.33
5-*Accurate listeners*	1	15	6.67	100.00

### Sum of Correct Answers by Gender

A significant issue was to check whether gender influences the listenability of sermons.

As can be seen in Table 7, there are no significant differences between men and women in listening to and not listening to sermons. The average number of correctly chosen answers, indicating an average attentive listening to a sermon, is almost equal, 3.19 for women and 3.12 for men. The value of *t* = 0.26, and the probability of *p* = 0.797 indicates that there is no statistically significant difference between men and women in listening to and not listening to sermons. This means that gender has no influence at all on whether or not a person listens to a sermon.

**Table 7:** Differences in listenability of sermons between men and women; Student's *t* test, female number = 82, male number = 48

Mean Females	Mean Males	Percentage of females	Percentage of males	SD Females	SD Males	*df*	*t* value	*p*
3.19	3.12	63.08	36.92	1.36	1.70	128	0.26	0.797

### Differences Between Churches

The survey was conducted with participants of masses in three churches, one rural parish and two city parishes. Therefore, there is a question whether there is a difference in the audience of sermons between rural and urban parishes.

As shown in Table 8, there are statistically significant differences between churches in terms of listening to sermons (*F* = 7.92, *p* = 0.001) with the highest (*M* = 3.68) in church number 2. In order to examine which differences are statistically significant, the a posteriori Scheffe test was used.

**Table 8:** Descriptive statistics of sermon listenability in the studied churches and ANOVA differences; 1 = rural parish, 2 and 3 = city parishes

Church	Mean	N	SD	SS	df	MS	F	p
1	3.23	30	1.17	31.75	2	15.87	7.92	0.001
2	3.68	53	1.36					
3	2.55	47	1.61					
Total	3.17	130	1.49					

Table 9 shows that there are statistically significant differences between churches 2 and 3 (*p *= 0.001), i.e. between two city churches; there are no statistically significant differences between city and rural churches. This means that the listenability of the sermons does not depend on whether the parish is urban or rural.

**Table 9:** Listenability of sermons in individual churches, Scheffe test indicates the significance of the differences

Church	1 *M *= 3.23	2 *M *= 3.68	3 *M *= 2.55
1		0.39	0.13
2	0.39		0.001
3	0.13	0.001	

### Listening to Sermons in the Population

It seems that the research conducted allows us to draw conclusions for the whole population, because the participants in the survey were a representative sample taken from the entire population, i.e. to answer the question how Catholics throughout the country listen to the sermons.

As can be seen in Table 10, the average listening rate for sermons is 3.17 and the standard deviation is 1.49, which means that people do not listen well to sermons. The confidence interval shows that the average value in the population, with a probability of error not exceeding 5%, is between 2.9 and 3.4, and hence it can be deduced that throughout Poland people do not listen very carefully to sermons (i.e. they listen on average accurately, moderately carefully or half-heartedly). Median 3.0 also confirms this. The mode is 4, but this represents only 28% of those who listen more carefully. To sum up, the confidence interval shows that the average general listenability of sermons in Poland is average, i.e. people do not listen to sermons very well.

**Table 10:** Basic characteristics of listening to sermons in the whole test sample, *N *= 130

Mean	Std. error of the mean	Confidence interval 95%	Median	Mode	Mode frequency	SD	Confidence interval SD 95%	Skewness	Kurtosis
3.17	0.13	2.91–3.43	3.0	4.0	37	1.49	1.33–1.70	− 0.68	− 0.32

### Listenability in Age Groups

The respondents were classified into age groups in the same way as before.

Descriptive statistics of the listenability of sermons in age group 16–39 show that mean value is 3.55 and SD = 1.30, what means that young people listen to sermons on average attentively, but slightly more accurately than the average in the population. As can be seen (Table 3), among the surveyed people the greatest number of people listened to the sermon on average accurately and quite attentively (over 27% of *Average Listeners* and 31% of *Fairly Accurate Listeners*, respectively).

In the next age group of 40–59 years (Table 11), the average listening rate of sermons is 3.65, standard deviation 1.32, which means that people listen to sermons accurately on average. However, in this group most people listened to the sermon fairly attentively (38% of *Fairly Accurate Listeners*, Table 4).

**Table 11:** Descriptive statistics of the listenability of sermons in age groups

Age (in years)	Mean	SD	Mode	Mode frequency	Median	*N*
16–39	3.55	1.30	4.0	16	4.0	51
40–59	3.65	1.32	4.0	13	4.0	34
60–79	2.50	1.57	2.0	8	2.5	30
80–92	2.13	1.41	2.0	7	2.0	15

Additional analysis showed that in the subgroup 40–49 years of age, there were nobody from *Non*-*Listeners* or even poor listeners, i.e. this is an age group that tends to listen attentively. Moreover, sermons are listened to most attentively, most often by people in the age group 30–49 (mean score of 4.0), which is a certain pastoral allusion to take this age group into account in sermons in particular, as they will benefit most from the sermon.

As can be seen in Table 11, among 60–79 year olds, the average listening rate for sermons is 2.5 and standard deviation is 1.57, which means that people do not listen carefully to sermons. These results are slightly lower than the population mean. Most of these people listened to the sermon with a little attention (almost 27% of *Low Accuracy Listeners*, Table 5).

However, for people over 79 years of age, the average listening rate of sermons is 2.1 and the standard deviation is 1.41, which means that people do not listen carefully to sermons. Most people listened to the sermon with little attention (almost 47% of *Low Accuracy Listeners*, Table 6). Nevertheless, it must be taken into account that this is an age group, in which short-term memory may also be slightly weaker, although on the other hand, it should be noted that among these people there were also people who listened very carefully.

### Declared and Actual Listening to Sermons

The relationship between the declared and actual listening to sermons by people seems to be particularly relevant in the context of this article.

As can be seen in Table 12, there is no significant correlation between the declared and actual listening of the sermons. The correlation coefficient is − 0.28, which means that 7.84% of the variance of the declared listening to sermons is explained by the variance of the actual listening to sermons (number of correct answers). This means that the variability of the declared answers concerning listening to the sermons explains only 7.84% of the variability of the answers indicating the actual listening to the sermons. Such a small correlation coefficient is statistically insignificant at the level of significance *p* > 0.05. Therefore, it can be concluded that the declared listening to sermons has no statistically significant relationship with the actual listening to them.

**Table 12:** Characteristics of declared and actual listenability of sermons and correlation between them; *N *= 47

Listenability	Mean	SD.	Declared	Actual	Significance
Declared	1.79	1.04	1.00	− 0.28	Not significant
Actual	2.55	1.61	− 0.28	1.00	Not significant

**Fig. 2:**
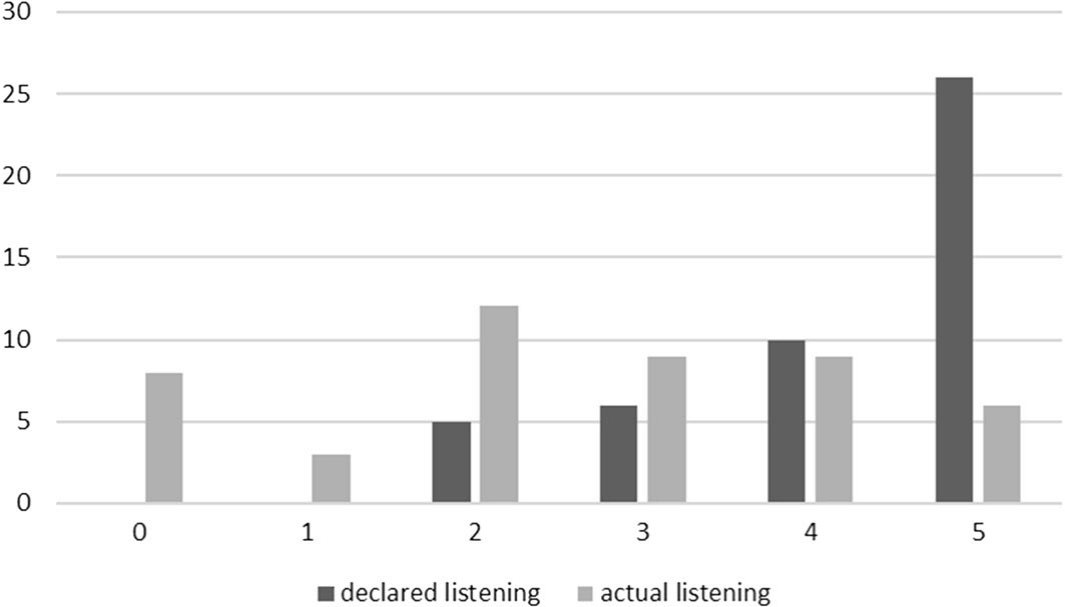
Declared listening to sermons. question: do you like to listen to sermons? Answers (horizontal axis): 5. yes, and I always listen to them to use them in my life; 4. yes, but I don’t always listen; 3. sometimes, I listen to some Priests; 2. sometimes, when there are special occasions; 1. no and I don’t listen; 0. no and I never listen. Actual listening to sermons: 5. accurate listeners, 4. fairly accurate listeners, 3. average listeners, 2. low accuracy listeners, 1. almost non-existent listeners, 0. non-listeners. Vertical axis: number of people. *N* = 47

Figure 2 clearly shows the difference between the declared and the actual listening of the sermons. The correlation coefficient is statistically insignificant (*r* = − 0.28, *p* > 0.05), which confirms the hypothesis that there is no correlation between the declared and actual listening of sermons. People who declare that they always listen to the sermons, in fact, only partially listen to the sermon (the number of correct answers indicates about 60% of the sermon). It can be concluded from this that people who declare themselves to be listening to the sermons and like to listen to them, in fact listen to them partially, in the range of about 50–70%, as indicated by the 95% confidence interval. On the other hand, people who declare that they like to listen to sermons, but do not always listen to them, actually listen to about 1/3 of the sermon (about 35%), and the 95% confidence interval indicates that in the population such people listen to the sermon from 10 to 55%. Besides, no one has declared that he or she does not listen to the sermons at all, although there were 9% of the people who did not listen to the sermons at all in reality.

From this, it can be concluded that research based solely on self-assessment and declarations such as “listen/do not listen” or “like/do not like” turns out not to give a true picture of whether the persons concerned are actually listening to the sermons.

## Summary

**Fig. 3:**
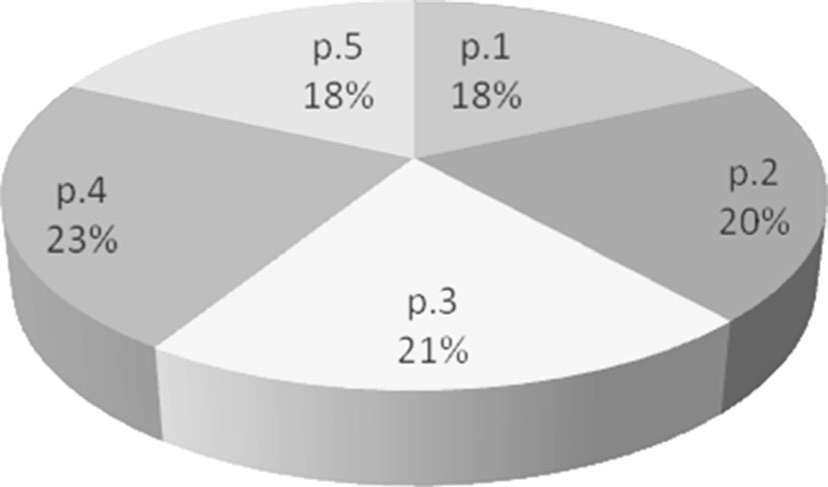
Percentage distribution of the listenability of particular parts of the sermon, p.1 to p.5 indicate each part of the sermon from beginning to end

Catholics usually don’t listen very carefully to sermons. Listening to a sermon does not depend on gender or whether the parish is urban or rural. Moreover, on the basis of the results of the research, it turns out (which confirms the initial assumptions of the research) that the “I am listening to the sermon” declarations are not covered by the actual listening to them (Table 12, Figs. 2, 3). Also, the “I liked”/”I didn’t like” declarations do not indicate whether the person listened to the sermon.

Most people listen to the central part of the sermon. The CKSK method makes it possible to conduct research whose results answer the question whether Catholics are listening to the sermons. However, preachers who wish to instruct their listeners about the truths of faith convince them to undertake specific tasks and the Christian lifestyle, may still ask the question whether the content prepared and preached by them is received, remembered, and implemented. The answer to this question, intended by the authors, will be the subject of further research.

The high hearing score at the end of the sermon, in the case of a well-prepared sermon (the sermons the subjects listened to were different; one was very well prepared, the other inappropriately, the third was medium), suggests “joining in”, i.e. to begin listening from a certain moment (later than the beginning), by interested listeners, thanks to a well-prepared (and not too short) sermon. People who are curious start to listen. This is a pastoral allusion to carefully prepare the sermons and not to neglect to write them. A written and well-prepared sermon will do its job because the faithful will be more willing to listen to it. Meanwhile, such sermons, which are built on merely repeating the words of Sacred Scripture, quoting, repeating with the language of a poet, historian, archaeologist, or even an exegete (like the third of the sermons), are merely repeating, literally, or paraphrasing, and actually give nothing to people, no explanation, no guidance or advice (Świerzawski 1964); their advantage is that they are only a reminder of Sacred Scripture. The problem is that sermons, which seem to forget God, resemble secular, and not very well-prepared speeches (like the second); it is likely that such sermons discourage people from attentive listening.

Whether Catholics listen to sermons is part of another question, namely, whether people listen to what is said to them. As you can see, sermons are listened to most carefully by people in the age group 30–49. This is a pastoral allusion to the preparation of sermons with a special emphasis on the 30–49 age group, because it will benefit the most from the sermon. Listening is essential for remembering and translating into concrete aspects of one’s own life. A listener who not only hear, but he or she does listen to the sermon can relate it to his own life. The art of listening to something is more than just hearing something. This translates into various aspects of life, e.g. in communication with others, “to do listen to” is the ability of the heart that enables closeness, without which there can be no real spiritual encounter (Pope Francis, 171).

In addition, for theologians and psychologists it is important to ask whether Catholics listen to sermons, and it may be a little disturbing that many people listen to sermons in a very fragmented way. On the one hand, meeting people with various illnesses, for whom faith is very helpful; on the other hand, trying to help people, for example, recruited to sects, who show a lack of knowledge of the principles of religion, the inability to distinguish between what is good and what is bad; then, among other things, there is a doubt whether nobody gave them hope for eternal life, did not explain the issues of faith, or rather they did not listen to it. The results of CKSK research suggest that people who do not listen to what priests have to say about their faith become more susceptible to recruitment of sects and other destructive groups, although this is only one of many aspects of vulnerability to spiritual threats (Noworol OV 2018).

The issues of how the quality of the sermon affects its listenability (influence of content, length of the sermon, listening from/to a certain moment, and listening to some priests), e.g. whether a sermon showing holiness and goodness would attract more listeners (which suggests the first of the sermons), whether a sermon telling secular rhymes and other not fully thought out attempts at “following the spirit of the times”, does not discourage listening, etc., are the authors intend to make in the future, as they will be the subject of further research.
